# Hospital-based Surveillance Provides Insights Into the Etiology of Pediatric Bacterial Meningitis in Yaoundé, Cameroon, in the Post-Vaccine Era

**DOI:** 10.1093/cid/ciz506

**Published:** 2019-09-05

**Authors:** Angeline Boula, Madikay Senghore, Rose Ngoh, Flaubert Tassadjo, Marie-Christine Fonkoua, Ariane Nzouankeu, Mina Kenkela Njiki, Jeanne Musi, Sandrine Bebey, Madeline Ngo Baleba, Angeline Nkembe, Sidonie Médjina, Peter S Ndow, Archibald Worwui, Marie Kobela, Marceline Nimpa, Jason M Mwenda, Aboubacar N’diaye, Brenda A Kwambana-Adams, Martin Antonio

**Affiliations:** 1 Centre Mere et Enfant de la Fondation, Yaoundé, Cameroon; 2 World Health Organization (WHO) Collaborating Centre for New Vaccines Surveillance, West Africa Partnerships and Strategy, Medical Research Council Unit The Gambia at London School of Hygiene and Tropical Medicine, Banjul, The Gambia; 3 Centre de Pasteur, Yaoundé; 4 Expanded Programme on Immunization, Yaoundé; 5 WHO Country Office Cameroon, Yaoundé; 6 WHO Regional Office for Africa, Brazzaville, Republic of Congo; 7 Microbiology and Infection Unit, Warwick Medical School, University of Warwick, Coventry, United Kingdom

**Keywords:** pediatric bacterial meningitis, conjugate vaccine, genotyping

## Abstract

**Background:**

Meningitis is endemic to regions of Cameroon outside the meningitis belt including the capital city, Yaoundé. Through surveillance, we studied the etiology and molecular epidemiology of pediatric bacterial meningitis in Yaoundé from 2010 to 2016.

**Methods:**

Lumbar puncture was performed on 5958 suspected meningitis cases; 765 specimens were further tested by culture, latex agglutination, and/or polymerase chain reaction (PCR). Serotyping/grouping, antimicrobial susceptibility testing, and/or whole genome sequencing were performed where applicable.

**Results:**

The leading pathogens detected among the 126 confirmed cases were *Streptococcus pneumoniae* (93 [73.8%]), *Haemophilus influenzae* (18 [14.3%]), and *Neisseria meningitidis* (15 [11.9%]). We identified more vaccine serotypes (19 [61%]) than nonvaccine serotypes (12 [39%]); however, in the latter years non–pneumococcal conjugate vaccine serotypes were more common. Whole genome data on 29 *S. pneumoniae* isolates identified related strains (<30 single-nucleotide polymorphism difference). All but 1 of the genomes harbored a resistance genotype to at least 1 antibiotic, and vaccine serotypes harbored more resistance genes than nonvaccine serotypes (*P* < .05). Of 9 cases of *H. influenzae*, 8 were type b (Hib) and 1 was type f. However, the cases of Hib were either in unvaccinated individuals or children who had not yet received all 3 doses. We were unable to serogroup the *N. meningitidis* cases by PCR.

**Conclusions:**

*Streptococcus pneumoniae* remains a leading cause of pediatric bacterial meningitis, and nonvaccine serotypes may play a bigger role in disease etiology in the postvaccine era. There is evidence of Hib disease among children in Cameroon, which warrants further investigation.

## Teaser

We studied the etiology of pediatric bacterial meningitis in Cameroon, in the postvaccine era, associated with *Streptococcus pneumoniae*, *Haemophilus influenzae*, and *Neisseria meningitidis.* Our findings emphasize the need for increased efforts to alleviate vaccine-preventable disease in our subregion.

Bacterial meningitis causes serious illness in children, which can lead to death and permanent neurological sequelae in survivors [1]. Meningitis is endemic to regions of Cameroon that lie outside the meningitis belt, including the capital city, Yaoundé. A study conducted in Yaoundé between 1999 and 2000 found that the majority of bacterial meningitis cases were due to *Streptococcus pneumoniae* (56.2%), *Haemophilus influenzae* (18.5%), and *Neisseria meningitidis* (13.4%) and noted that children aged <5 years were the most vulnerable age group [2]. In 2013, Nguefack and colleagues carried out a study at the Yaoundé Gyneco-obstetric and Pediatric Hospital to investigate the etiology and outcomes of children with purulent meningitis [3]. Their study enrolled 171 children aged <15 years with confirmed bacterial meningitis by culture or antigen test, most of whom were <5 years of age (141 [82.5%]). More than 10 species were reported to cause meningitis, the leading causes being *H. influenzae* in 67 children (39.2%), followed by *S. pneumoniae* in 54 children (31.6%) and *N. meningitidis* in 17 children (9.9%) [3]. A high number of deaths and neurological sequelae were reported: 32 deaths (18.7%) and 17 cases of sequelae (9.9%) [3].

In the past decade, important vaccine interventions have targeted the main pathogens causing meningitis in Cameroon. The *H. influenzae* type b (Hib) conjugate vaccine was introduced in 2009 and since then the coverage has remained >80% [4]. In 2011, the 13-valent pneumococcal conjugate vaccine (PCV13) was introduced into the Cameroon Expanded Programme on Immunization. Although the estimated coverage of children immunized with PCV13 was low (23%) in 2011, it rose to 85% in subsequent years. In December 2011, Cameroon became the fourth country in the meningitis belt to roll out the meningococcal A conjugate vaccine (MenAfriVac), which targets *N. meningitidis* serogroup A, on a large scale. The efficacy of PCV13, Hib vaccine, and MenAfriVac in reducing the burden of disease in resource-poor settings is well documented [5–9]. However, in Cameroon little is known about how the etiology of pediatric bacterial meningitis (PBM) has changed, if at all, following these interventions.

Whole genome sequencing (WGS) has emerged as a powerful tool for genotyping and tracking the evolution of pathogenic bacteria. By comparing genome sequences to curated databases, we can scan for known antibiotic resistance and virulence genotypes. Incorporating genomic data into ongoing surveillance allows early detection of emerging virulent clones that can potentially lead to meningitis outbreaks [10]. WGS allows us to discriminate between genotypes within the same serotype and provide insights into the evolution of bacterial pathogens [5].

In 2002, the World Health Organization (WHO) set up the global surveillance network for invasive bacterial vaccine-preventable diseases (IB-VPD), to accurately describe the burden and epidemiology of IB-VPD and to assess the impact of vaccines on disease burden and epidemiology, particularly the Hib vaccine and pneumococcal conjugate vaccines [6]. The WHO Collaborating Centre (CC) for New Vaccines Surveillance, hosted at the Medical Research Council Unit The Gambia (MRCG), supports the IB-VPD surveillance network for West and Central Africa in 10 countries including Cameroon.

Here, we present findings on the etiology and molecular epidemiology of meningitis in Cameroon’s capital city from 2010 through 2016. We assessed the temporal changes in the pathogens detected from suspected cases of meningitis and used molecular techniques to further describe their epidemiology including WGS on a subset of *S. pneumoniae* isolates.

## METHODS

### Study Site

Bacterial meningitis surveillance is ongoing at the Centre Mere et Enfant de la Fondation Chantal Biya (CME) hospital, Yaoundé, which is approximately 600 km south of the meningitis belt. The CME is a specialist hospital that serves as the main pediatric referral hospital in Cameroon. Yaounde has an equatorial rain forest climate and is not adversely affected by the harmattan winds that prevail in the dry season.

### Patients

This surveillance was conducted among children <5 years old. Suspected meningitis was defined as sudden onset of fever (>38.5°C rectal or 38.0°C axillary) accompanied by 1 or more of the following symptoms: neck stiffness, altered consciousness, or hypersensitivity to light. Demographic data including age, sex, region of residence, vaccination history, and prior antibiotic use was recorded on a case investigation form. Clinical information regarding admission date, diagnosis, outcome, comorbidities, and sequelae was also recorded.

### Study Specimens

Unless clinically contraindicated, cerebrospinal fluid (CSF) specimens were collected from children enrolled in the surveillance. Each specimen was linked to a case investigation form by a unique identification number. CSF specimens were visually inspected and the appearance of the CSF specimens was recorded. Serological tests including white blood cell (WBC) counts and biochemical tests for glucose and protein levels were also performed when available [7]. Suspected cases were classified as probable if the CSF was positive from Gram stain and/or appeared turbid or had a WBC count >100 cells/μL, or if the WBC count ranged between 10 and 100 cells/μL and CSF protein was >100 mg/dL or if the WBC count ranged between 10 and 100 cells/μL and CSF glucose was >40 mg/dL. Meningitis cases (n = 126) were confirmed for CSF specimens that had a pathogen detected by culture, rapid diagnostic test (Pastorex or Binax), or real-time polymerase chain reaction (PCR).

Pathogen detection by culture, latex agglutination, and/or PCR was performed on CSF from 765 suspected cases of meningitis. At the sentinel site in Yaoundé, 555 samples were tested by culture and/or latex: culture was performed on 500 (8.4%) suspected cases, whereas rapid testing was done using the Pastorex kit on 304 (5.1%) suspected cases. A total of 344 CSF specimens were sent to the WHO CC for pathogen detection and where possible, serotyping or serogrouping using quantitative PCR (qPCR).

### Molecular Characterization at WHO Collaborating Centre

Where sufficient volumes were available, CSF specimens were sent to the WHO CC hosted at MRCG for molecular characterization using PCR. In 2011 and 2012, only culture-negative CSF specimens were sent to the WHO CC for PCR; however, between 2013 and 2016 all categories of CSF specimens were sent to the WHO CC. During this period, 35 isolates of *S. pneumoniae* were sent to the WHO CC, based on availability and viability, and WGS was successfully performed on 29 isolates.

In brief, DNA was extracted from the CSF using a modified Qiagen extraction method as previously described [10]. A qPCR assay was used to detect the 3 main pathogens, *S. pneumoniae*, *N. meningitidis*, *and H. influenzae* targeting the *lytA*, *sodC*, and *hpd* genes, respectively. Serogrouping for *N. meningitidis* and *H. influenzae* was performed by qPCR for targeted serotype-specific genes as previously described [8]. *Streptococcus pneumoniae* serotyping was performed by subjecting samples to serial triplex PCR assays targeting 21 of the most common serotypes globally as previously described [9].

### Antimicrobial Susceptibility Testing and WGS

Isolates received at the WHO CC were subcultured onto gentamicin blood agar and cultured overnight at 37°C. Isolates were tested for susceptibility to the following antibiotics using the disk diffusion method, and inhibition zones were interpreted according to Clinical and Laboratory Standards Institute guidelines [11]: rifampicin, erythromycin, ceftriaxone/cefotaxime, tetracycline, vancomycin, oxacillin, chloramphenicol, trimethoprim-sulfamethoxazole, meropenem, and clindamycin.

Genomic DNA was extracted from fresh overnight cultures using a modified Qiagen kit protocol [10] and sent to the Wellcome Trust Sanger Institute for paired-end sequencing on an Illumina HiSeq [12]. Sequencing reads from each isolate were mapped onto the *S. pneumoniae* ATCC 700669 serotype 23F reference genome using SMALT [13], and pseudogenomes were placed in a multiple sequence alignment using custom scripts. Single-nucleotide polymorphisms (SNPs) were called from the pseudoalignment using SNP-sites, and a maximum likelihood phylogeny was reconstructed with a general time-reversible model using RAxML [14]. The phylogenetic tree was visualized and annotated using iTOL [15]. ARIBA was used to scan the genome for known resistance mutations and virulence genes [16]. We analyzed of 29 *S. pneumoniae* genomes isolated from confirmed meningitis cases.

### Ethical Considerations

Ethical approval was not a requirement in Cameroon for routine meningitis surveillance, including drug susceptibility testing of collected isolates, as surveillance is part of the approved routine diagnostic algorithm at the Ministry of Health. However, informed consent was sought from the parents or guardians of the surveillance participants. Additionally, the surveillance received overarching ethical approval (SCC1188) by the joint Medical Research Council (MRC)/The Gambia government ethics board that allowed the analysis of collected West African isolates at MRCG.

## RESULTS

### Patient Characteristics

A total of 5958 children <5 years of age with suspected cases of meningitis were recruited into the surveillance from the sentinel site in Yaoundé between 2010 and 2016. Nearly two-thirds of the patients admitted were in their first year of life and more than half were male (Table 1). The outcome at discharge was recorded for 2940 patients, and 141 (4.8%) were confirmed to have died before discharge. Lumbar puncture was performed by clinicians on all suspected cases to collect CSF. Most CSF samples (4165 [69.9%]) had clear appearance and WBC count <10 cells/μL (Table 1).

**Table 1.** Summary of Patient Information for All Children Admitted With Suspected Meningitis, 2010–2016

Characteristic	n	(%)
Age, mo		
0–11	3702	(62)
12–23	912	(15)
24–59	1343	(23)
Unknown	1	(0)
Sex		
Male	3326	(56)
Female	2632	(44)
Antibiotic before admission		
Yes	667	(11)
No	4889	(82)
Unknown	402	(7)
Case type^a^		
Suspected	5832	(98)
Confirmed	126	(2)
CSF appearance		
Clear	4165	(70)
Turbid	263	(4)
Xanthrochromic	824	(14)
Other	706	(12)
CSF WBC count, cells/μL		
<10	5235	(88)
10–100	490	(8)
>100	231	(4)
Unknown	2	(0)

Abbreviations: CSF, cerebrospinal fluid; WBC, white blood cell.

^a^Suspected cases include cases that were defined as probable as per World Health Organization case definition guidelines [17].

### Trends in Pathogen Detection by Culture, Rapid Test, and PCR Over Time

**Figure 1.**
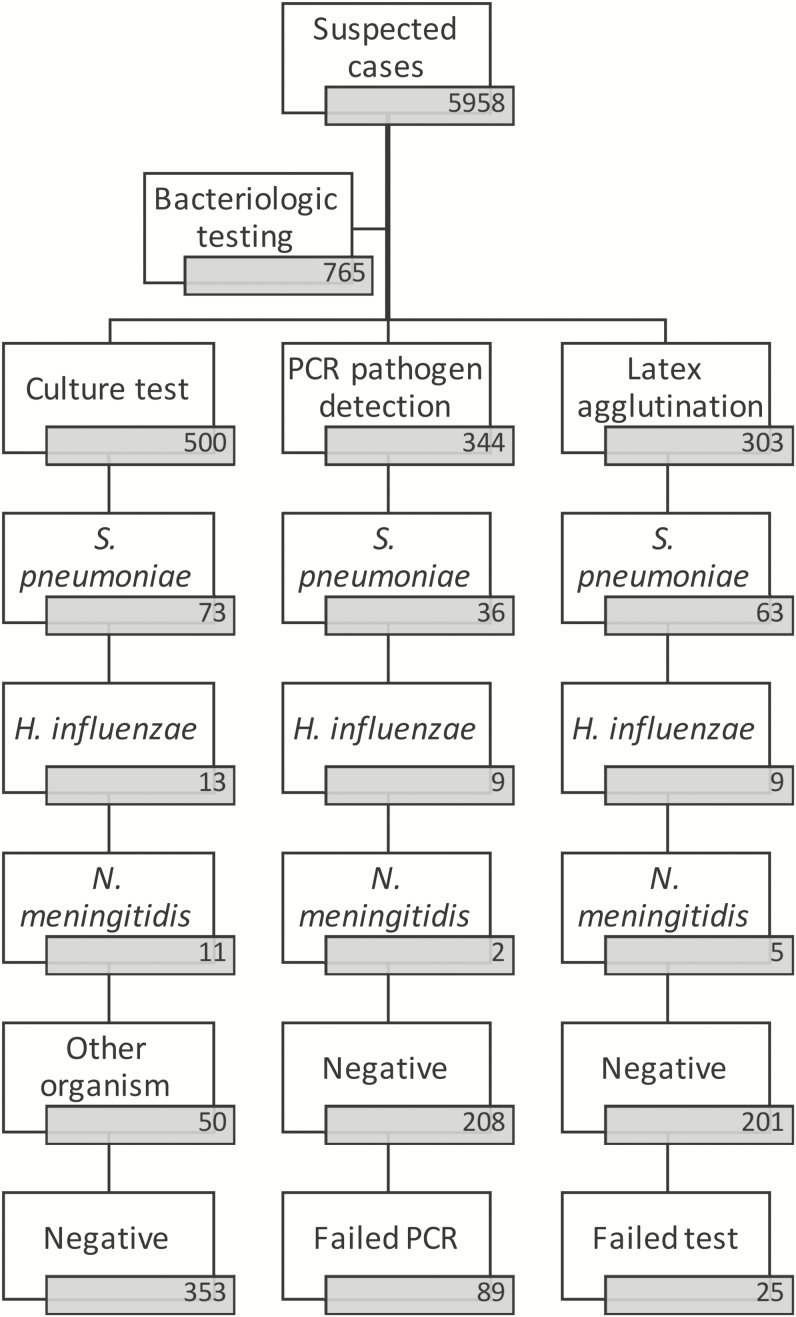
Flowchart summarizing the number of patients, how many had specimens tested by each of the 3 bacteriological methods, and the outcome of the tests. Abbreviation: PCR, polymerase chain reaction.

Subsets of the specimens were tested using culture, rapid tests, and/or PCR (Table 2), and we confirmed a total of 126 (16.5%) cases of bacterial meningitis. The leading pathogen detected among the 126 confirmed cases was *S. pneumoniae* (93 [73.8%]), followed by *H. influenzae* (18 [14.3%]) and *N. meningitidis* (15 [11.9%]) (Figure 1). Among 93 confirmed cases of *S. pneumoniae* meningitis, data on pneumococcal vaccination history were retrieved from the vaccination card or medical records of 35 (37.6%) infants; in most cases (54 [58.1%]), data were acquired through oral attestation and in 4 cases there was no record. Only 20 (21.5%) infants had confirmed receiving the pneumococcal conjugate vaccine, 54 (58%) did not report receiving the vaccine, and the vaccination history of 19 (20.5%) patients was unknown. Among the cases of *S. pneumoniae* meningitis that were vaccinated, 6 cases (30%) were associated with PCV13 serotypes.

**Table 2.** Patient Age and Sex for the Specimens That Were Tested by Each of the 3 Bacteriological Methods

Characteristic	Culture	PCR	Latex
Sex			
Female	214 (43)	140 (41)	130 (43)
Male	286 (57)	204 (59)	173 (57)
Age group, mo			
0–11	344 (69)	218 (63)	212 (70)
12–23	66 (13)	49 (14)	38 (13)
24–59	90 (18)	77 (23)	53 (17)

Data are presented as No. (%).

Abbreviation: PCR, polymerase chain reaction.

**Figure 2.**
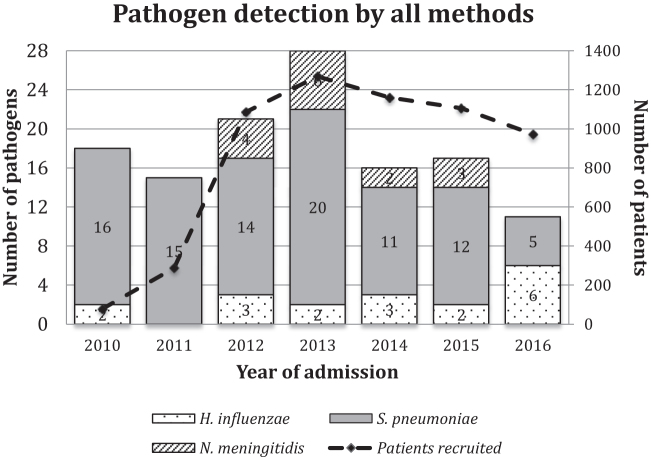
Bar chart showing the proportion of cases due to the 3 vaccine-preventable pathogens detected by a combination of culture, rapid tests, and polymerase chain reaction. Secondary axis shows the total number of patients recruited in each year. The *Haemophilus influenzae* type B vaccine was introduced in 2009 and the 13-valent pneumococcal conjugate vaccine was introduced in 2011.

Over time, the proportion of cases that was attributed to each of the 3 main pathogens varied. Between 2010 and 2015, *S. pneumoniae* represented at least 60% of all positive cases of bacterial meningitis, but this dropped to <50% in 2016. The proportion of cases due to *H. influenzae* was low up to 2015 but increased in 2016. This was due to a slight increase in the case count of *H. influenzae* and a sudden decrease in *S. pneumoniae* cases in 2016 (Figure 2). Low numbers of *N. meningitidis* cases were found between 2012 and 2015, but there were no confirmed cases of *N. meningitidis* meningitis in 2016.

### Molecular Characterization of Etiology and Serotyping

**Figure 3.**
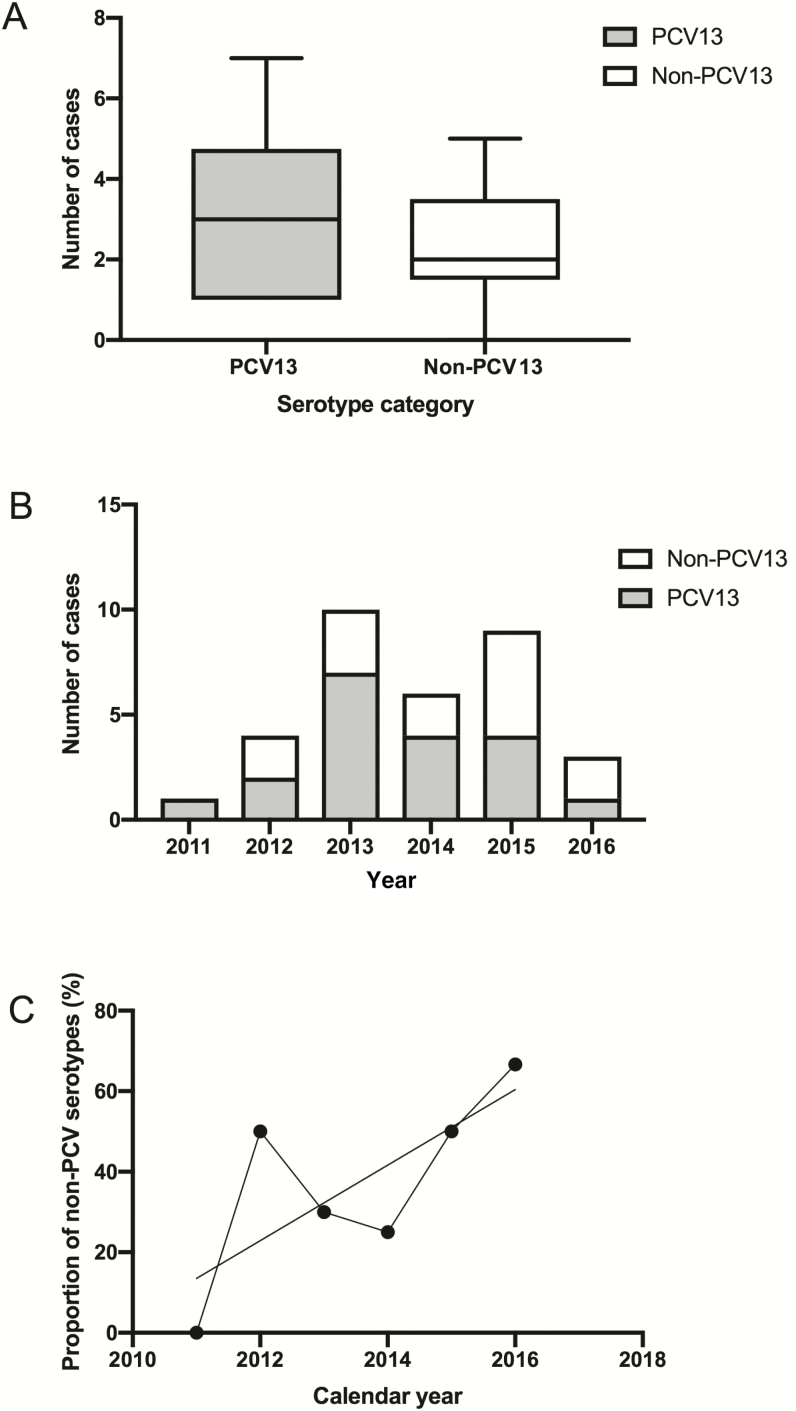
Distribution of the 13-valent pneumococcal conjugate vaccine (PCV13) and non-PCV13 serotypes over the surveillance period. *A*, Boxplot comparing the overall case counts of PCV13 and non–pneumococcal conjugate vaccine (PCV) serotypes based on the per annum counts. *B*, Stacked bar chart comparing the case counts per annum for PCV13 serotypes and non-PCV13 serotypes. *C*, Line graph of the proportion of PCV serotypes over time (years) with a nonlinear regression line of best fit.

The number of cases of *S. pneumoniae* detected by PCR fluctuated between 2011 and 2016. Using qPCR, we successfully serotyped 31 of 36 *S. pneumoniae* isolates that were detected by PCR. Between 2011 and 2016, PCR serotyping confirmed 19 and 12 cases of meningitis associated with PCV13 and non-PCV13 serotypes, respectively (Figure 3A). The numbers were generally low, but from 2013 to 2016 the proportions of non-PCV13 serotypes increased marginally (Figure 3B and 3C).

Nine *H. influenzae* strains were confirmed by PCR and typed by qPCR, of which 8 were Hib and 1 was type f. Among 8 patients that had Hib detected in their CSF, 1 had an unknown vaccination history, 5 were not vaccinated, and 2 were vaccinated but had not completed the full 3-dose regimen: 1 patient had received 1 dose and 1 had received 2 doses of the vaccine. Both patients were females admitted with suspected meningitis in 2014 aged 2 and 4 months old, respectively. The 2 cases of *N. meningitidis* detected by PCR could not be serogrouped by qPCR.

### Whole Genome Phylogeny of *S. pneumoniae*

**Figure 4.**
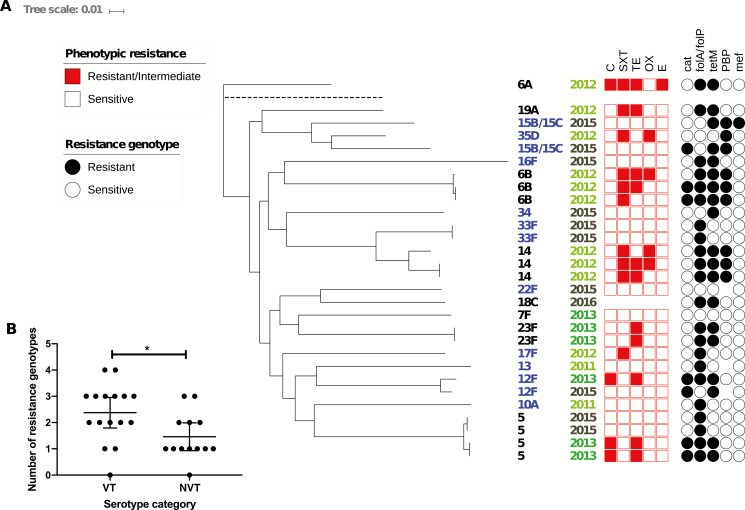
*A*, Maximum likelihood whole genome phylogenetic tree of *Streptococcus pneumoniae* isolates recovered from cerebrospinal fluid. *B*, Column graph comparing the number of resistance genotypes per genome among vaccine serotypes (VT) and nonvaccine serotypes (NVT). In the phylogeny, the serotypes in blue are nonvaccine types and black are vaccine types; earlier years are shaded in a brighter shade of green; resistance/intermediate resistance to chloramphenicol (C), trimethoprim-sulfamethoxazole (SXT), tetracycline (TE), oxacillin (OX), and erythromycin (E) is shown by a red box; and the presence of the antibiotic resistance genes *catQ* (chloramphenicol), *folA/ folP* (trimethoprim), *tetM* (tetracycline), penicillin-binding proteins (PBP; penicillin), and *mef* (erythromycin) is shown by a black circle. Blanks mean no data or not tested. Reference genome is shown on the tree branch with dashed line.

The phylogeny of the *S. pneumoniae* isolates unearthed examples of strains that were very similar to one another (<30 SNP difference), as shown by the flat branches in the tree (Figure 4). This was more common with vaccine serotypes such as 5, 6B, 14, and 23F, but was also seen in the nonvaccine serotype 33F. In all cases, the similar strains both caused meningitis in the same year. For example, the 4 serotype 5 isolates, which were all ST289, formed 2 subclades, each with 2 isolates. The 2 isolates from 2013 differed by 19 core genome SNPs, whereas the 2 from 2015 differed by 15 core genome SNPs. In the genome-sequenced subset, between 2011 and 2013 the proportion of nonvaccine serotypes was 28% compared with 73% in 2015 and 2016 (Figure 3C).

Phenotypic resistance to chloramphenicol (n = 4 [13.8%]), trimethoprim-sulfamethoxazole (n = 10 [34.5%]), tetracycline (n = 10 [34.5%], plus 1 intermediate resistance), and oxacillin (n = 4 [13.8%]) was shown, and 1 isolate showed intermediate resistance to erythromycin. The WGS analysis revealed resistance genotypes in isolates that were classed as sensitive by disk diffusion. All but 1 of our isolates harbored a resistance genotype to at least 1 antibiotic. No known virulence genes were found by ARIBA in this dataset.

Interestingly, isolates collected between 2011 and 2013 had on average a higher number of resistance genotypes per genome (2.3) compared with isolates collected in 2015 and 2016, which averaged 1.5 per genome. Similarly, nonvaccine serotypes averaged a significantly lower number of resistance genes—on average, 1.5 resistance genotypes per genome compared with 2.4 per genome in vaccine serotypes (unpaired *t* test: *P =* .0212, difference of means [nonvaccine type – vaccine type], –0.9135 ± 0.3733 [95% confidence interval, –1.679 to –.1476]) (Figure 4B). This was exemplified by serotype 5 where the 2013 genomes bore 3 resistance genotypes to chloramphenicol, trimethoprim, and tetracycline, but the 2015 genomes only had the trimethoprim resistance genotype. Vaccines serotypes such as 6B and 14 from 2012 also bore at least 3 resistance genotypes.

## DISCUSSION

Meningitis continues to be a cause of serious illness and death among children in Cameroon, and we show that vaccine-preventable forms of the disease continue to play a role. Despite the introduction of PCV13, our study highlighted *S. pneumoniae* as the main etiologic pathogen causing PBM in Yaoundé. The low numbers in the study prevent us from drawing definitive conclusions on the effect of PCV13. However, both the PCR and genomics data showed early signs that nonvaccine serotypes may expand and play a more important role in causing disease in the post-PCV13 era. This phenomenon, known commonly as serotype replacement, is a common occurrence that follows introduction of conjugate vaccines [18–21]. Our genomics dataset further suggests that the expansion of nonvaccine serotypes may be accompanied by a welcome decline in the prevalence of antibiotic resistance genotypes borne within the *S. pneumoniae* genomes. Vaccines have been proposed as a means of decreasing antibiotic resistance, but it is too early to tell whether that may be the case in Cameroon [22–24].

Annually, the number of confirmed cases of *H. influenzae* and *N. meningitidis* remained low throughout the study period. Half of the *H. influenzae* cases were serotyped, and almost all were Hib. The confirmed cases of Hib were linked to either unvaccinated individuals or individuals who were yet to complete the full course of 3 vaccine doses. This highlights the importance of ensuring full coverage of the Hib vaccine among young children, but also raises concerns regarding the vulnerability of young infants who have not been fully vaccinated with all 3 doses of the Hib vaccine. Generally the introduction of the Hib vaccine in low-resource settings of Africa and Asia has proven to be an effective means to reduce the burden of Hib invasive disease among children <5 years of age [25, 26].

*Neisseria meningitis* has been known to cause meningitis in Cameroon for a long time. Outbreaks of meningitis have been caused by *N. meningitidis* serogroup A in the past [27, 28]. Moreover, around the year 2000 there was a surge in *N. meningitidis* meningitis cases due to an increase in serogroup A and the emergence of serogroup W135 [29]. Unfortunately, we were not able to serotype the *N. meningitidis* meningitis cases in our study, so we could not compare our results to past trends.

Our analysis provides baseline information into the genomic epidemiology of *S. pneumoniae* causing pediatric meningitis in Yaoundé and provides insights into the phylogeny of *S. pneumoniae* in this setting. The small size of the genomic dataset makes it difficult to infer definitive trends. Improved microbiology protocols and practices may improve isolate recovery rates and allow us to generate a more representative genomic dataset. Concerted efforts need to be made to ensure that children are adequately protected from PBM in Cameroon and in other low-income settings where the disease is endemic.

There were limitations to this study. In 2010 and 2011, the earlier years of surveillance, the patient recruitment was comparatively low. The surveillance team worked together to improve case reporting, reflected by a subsequent increase in patient recruitment. Moreover, the ratio of samples collected vs the number of samples tested was low: Only 765 (12.8%) samples collected were tested for pathogen detection. The number of unknowns regarding sequelae and treatment outcome was high and the form of sequelae was not recorded, making it difficult to study the long-term adverse effect of meningitis in infants.

## CONCLUSIONS

We present findings on the etiology of PBM in Yaoundé, Cameroon. *Streptococcus pneumoniae* remains a leading cause of meningitis in Yaoundé among children <5 years of age. The annual case counts of *H. influenzae and N. meningitidis* were low during the study period. Our data demonstrate the added value of PCR techniques for pathogen detection and molecular serotyping/serogrouping.

## Notes

***Invasive Bacterial Disease (IBD) Writing Group members.*** Brenda Kwambana-Adams, Madikay Senghore, Effua Usuf, Archibald Worwui, Egere Uzochukwu, Akram Zaman, Catherine Okoi, Florian Gehre, Leopold Tientcheu, Nuredin Ibrahim Mohammed, Felix Dube, Peter Ndow, Sambou Suso, Sheikh Jarju, Dam Khanx, Ebruke Chinelo, Rowan Bancroft, Jason M. Mwenda, and Martin Antonio.

***Author contributions.*** M. A. and J. M. M. established the World Health Organization (WHO) Regional Office for Africa (AFRO)–supported Paediatric Bacterial Meningitis Surveillance Network in West Africa. M. A. supervised the overall network including setting up the sentinel surveillance system. A. B., F. T., M. C. F., A. N., M. N., L. M., E. S., and J. N. clinically investigated and recruited the patients at the sentinel sites, collected demographic data and performed microbiological testing at sentinel sites, and shipped cerebrospinal fluid and bacterial isolates to the WHO Collaborating Centre (CC) at Medical Research Council Unit The Gambia (MRCG) for confirmatory testing and molecular analysis, supervised by B. K. A. and M. A. In addition, B. K. A. and M. A. developed the analysis plan and contributed to analysis and interpretation of data along with the IBD writing group. M. S., B. K. A., and M. A. drafted the manuscript along with A. B. All authors contributed to the interpretation of the findings and the writing of the final manuscript.

***Acknowledgments.*** The authors thank the WHO Country Office in Cameroon and the WHO Intercountry Support Team for coordination, advice, and support throughout the project; the surveillance participants and their families in Cameroon; and the staff and students at the WHO CC at the MRCG, as well as the IBD writing group for their advice and input.

***Financial support.*** Financial support for sentinel site surveillance was provided by the Cameroon Ministry of Health, Gavi - the Vaccine Alliance, through a grant to the WHO for the African Paediatric Bacterial Meningitis Surveillance Network.

***Supplement sponsorship.*** This supplement was supported with funds from Gavi, the Vaccine Alliance through The World Health Organization and the CDC Foundation, and The Medical Research Council Unit The Gambia at the London School of Hygiene and Tropical Medicine.

***Potential conflicts of interest.*** The authors reported no conflicts of interest. All authors have submitted the ICMJE Form for Disclosure of Potential Conflicts of Interest. Conflicts that the editors consider relevant to the content of the manuscript have been disclosed.
